# Retraction: Transcriptome study of receptive endometrium in overweight and obese women shows important expression differences in immune response and inflammatory pathways in women who do not conceive

**DOI:** 10.1371/journal.pone.0339145

**Published:** 2025-12-18

**Authors:** The PLOS One Editors

The *PLOS One* Editors retract this article [1] because it was identified as one of a series of submissions for which we have concerns about compromised peer review and compliance with PLOS policy on Manipulation of the Publication Process.

VS, EVB, and TBP did not agree with the retraction. AM either did not respond directly or could not be reached.
